# Root order-based traits of Manchurian walnut & larch and their plasticity under interspecific competition

**DOI:** 10.1038/s41598-018-27832-0

**Published:** 2018-06-29

**Authors:** Salahuddin, Boris Rewald, Muhammad Razaq, Yang Lixue, Ji Li, Farmanullah Khan, Zhang Jie

**Affiliations:** 1 School of Forestry, Northeast Forestry University, Harbin, China; 2 Agricultural Research Institute, Mingora, Pakistan; 3 Department of Forest and Soil Sciences, University of Natural Resources and Life Sciences (BOKU), Vienna, Austria; 4 Department of soil and environmental science, The university of Agriculture, Peshawar, Pakistan

## Abstract

Manchurian walnut and larch are key timber species of northeast China but information on (fine) root traits of both species is scarce. Plasticity of root traits in mixed plantations has been studied rarely although this could give important insights into mechanisms of root competition. This study examined root traits by branching order in 30-yr-old monocultures and their plasticity in mixed plantations. In monocultures, Manchurian walnut and larch differed in key fine root traits. Larch roots hold more absorptive root orders, larger diameter and lower specific root length/area. Walnut root orders featured greater cortex:stele ratios, N-concentrations and respiration rates. Under interspecific competition, the proportion of walnut root tips increased, the biomass/length of larch root orders 1–3 decreased. Larch possessed a greater morphological and anatomical plasticity of terminal root orders than walnut. Mycorrhizal colonization rates of walnut were reduced. Both species differed fundamentally in their fine root properties. Absorptive fine root orders reacted plastic under interspecific competition while traits of higher root orders remained unchanged. In mixture, larch roots possessed a greater plasticity in traits related to resource uptake (efficiency) than walnut roots whose reaction norm is suggested to be predominantly based on interference competition *via* juglone exudation.

## Introduction

Tree monocultures can provide large quantities of wood per unit area, however, this has often come at an expense, e.g. in terms of biodiversity, ecosystem system services and resilience in regard to climate change and biotic disturbances^1,2^. In contrast, mixed-species plantations may be less prone to such tradeoffs^3^, and may even provide increased production relative to monocultures^4–6^. The higher productivity of mixed-species plantations is the positive net result of negative (i.e. competition) and positive interactions (i.e. facilitation) among combined species including both above- and belowground interactions^7–9^.

Both Manchurian walnut (*Juglans mandshurica*) and larch (*Larix gmelinii*) are key timber species of northeast China and large plantations have been established in the last decades. While the productivity decline of long-term sites planted with Manchurian walnut monocultures remains a significant problem^10,11^, mixture with larch has been shown to stabilize or even increase the production^6,12,13^. Due to the economic importance, several previous studies have addressed the influence of larch on different aspects of Manchurian walnut seedlings to shed light on potential mechanisms involved in the inter-specific interactions. For example, extracts of larch root and bark, and root exudates were found to stimulate chlorophyll contents, soluble sugars and overall biomass of Manchurian walnut^14^. Larch root exudates increased the soil microbial biomass and enzymatic activities in mixed plantations with Manchurian walnuts^6,11^. In contrast, Manchurian walnut roots produce a highly phytotoxic exudate, juglone (5-hydroxy-1,4-naphthoquinone)^15,16^; juglone exerts an auto-inhibitory effect on seedlings^6^ and is likely to be more persistent in the soil of Manchurian walnut monocultures (due to the higher microbial biomass facilitated by larch)^17^.

Fine roots play a key role for carbon and nutrient cycles in terrestrial ecosystems^18,19^ and ensure water and nutrient uptake for plant survival, growth, and seed production^20^. Fine roots of neighboring plants in a mutual soil volume compete for resources, either by exploitative competition (i.e. reduction of water and nutrients resources)^21^ or through interference competition (i.e. the release of allelopathic chemicals which directly inhibit growth and thus the access to resources)^9^. Competition below ground is believed to be at least equally intense as shoot competition^22,23^. Resource competition and allelopathic chemicals, such as juglone, have been shown to reduce the growth of neighboring roots and plants^24,25^, and to influence traits of competing root systems to different extends^26,27^. Plants may acclimate to competition below ground by alteration in root biomass, root architecture (e.g. branching frequency), morphology (e.g. specific root length), root anatomy (e.g. cortex:stele ratio), physiology (e.g. maintenance respiration) and potentially intensity of mycorrhizal symbioses to increase the cost/benefit ratio and extend of resource capture^28–30^.

In the last decade, it has become increasingly clear that root systems of perennial plants consist of individual units (“root orders”) with distinct traits^31,32^. Especially changes in traits of the 2–3 terminal root orders (i.e. “absorptive fine roots”)^31^ can be expected to be most sensitive indicators for changes in the nutrient and water uptake capacities of branched root systems^29^. However, while structural and functional differences between root orders have been studied in some species^19,32,33^, studies addressing the effects of competition on order-based root traits remain scarce in general and limited to morphological and chemical traits in specific^26,34^. Similar, previous studies addressing belowground competition focused on biomass, morphology and/or tissue chemistry of whole root branches^27,35^ but largely neglected anatomical and physiological traits and changes among individual root orders.

Because previous studies on root traits of Manchurian walnut and larch were largely carried out under controlled conditions and focused on seedlings, the first objective of this study was to determine fine root system properties of mature (30 years-old) Manchurian walnut and larch trees *in situ*. We hypothesize that (i) the different ecology of Manchurian walnut and larch trees is reflected by highly divergent root traits in monocultures. Beyond the scope of previous studies, a wide range of root traits (architectural, morphological, anatomical, root respiration and mycorrhizal colonization rates) was studied across five terminal root orders. The second objective was to determine the plastic acclimation of both species’ fine roots systems to long-term interspecific competition. We hypothesize that (ii) the first 2–3 terminal root orders (i.e. “absorptive fine roots”) will react most plastic to interspecific competition while traits of higher root orders (i.e. “transport fine roots”) possess less adaptive plasticity. We hypothesize further, that (iii) larch roots will possess a greater plasticity in the studied traits (related to C use and resource uptake (efficiency)), under interspecific competition than Manchurian walnut roots whose reaction norm is suggested to be predominantly based on interference competition *via* juglone exudation.

## Results

### Root architecture, morphology and anatomy

**Figure 1:**
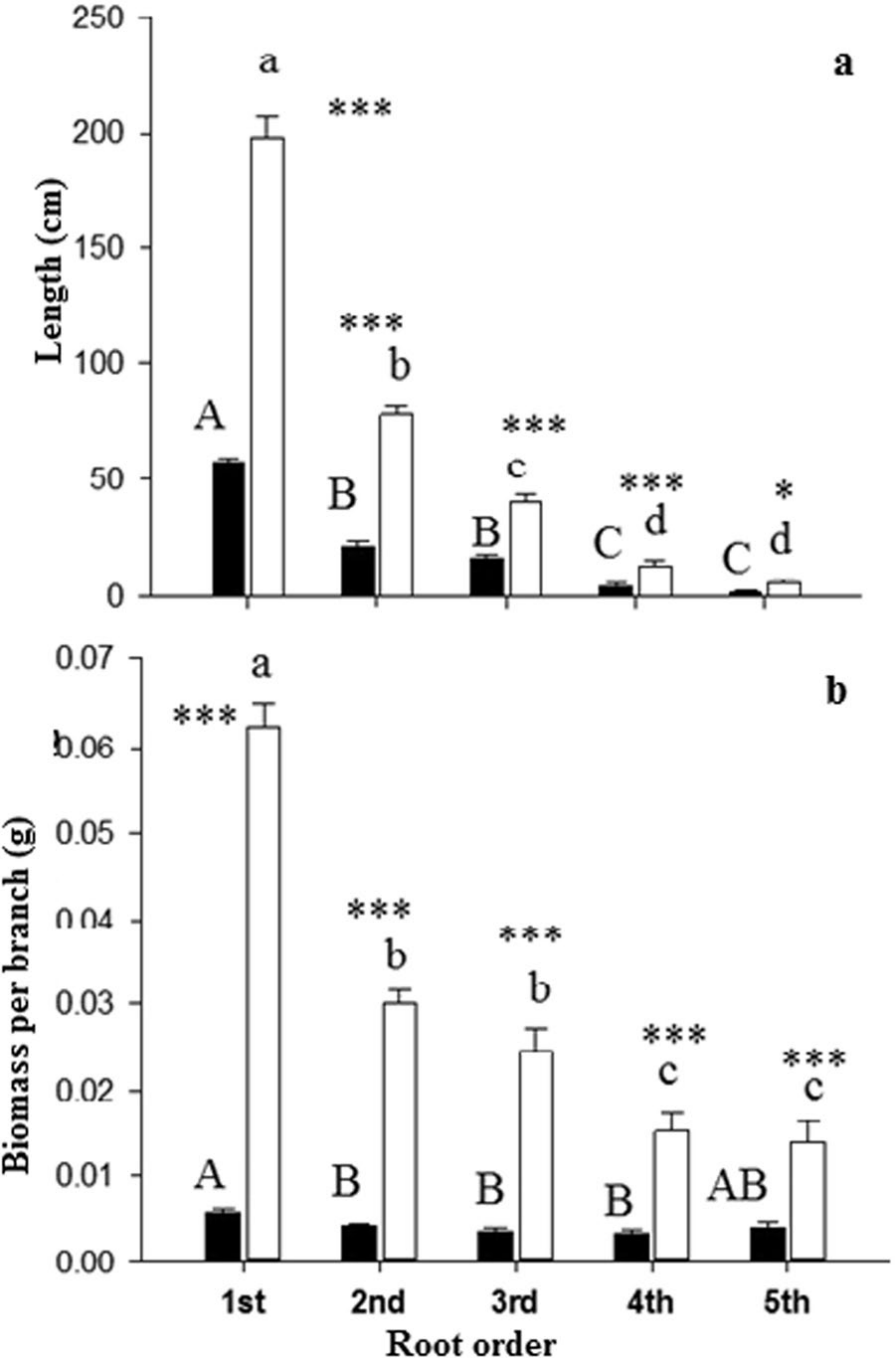
Root architecture (**a**,**b**) of root orders 1–5 of Manchurian walnut (black) and larch (white). Different upper case letters indicate significant differences between root orders of Manchurian walnut, lower case letters for larch (P < 0.05, Tukey HSD). Significant differences in a given root order class between species are marked with an asterisks (^*^P < 0.05; ^***^P < 0.001, Tukey HSD; n = 10–16; mean ± SE).

**Figure 2:**
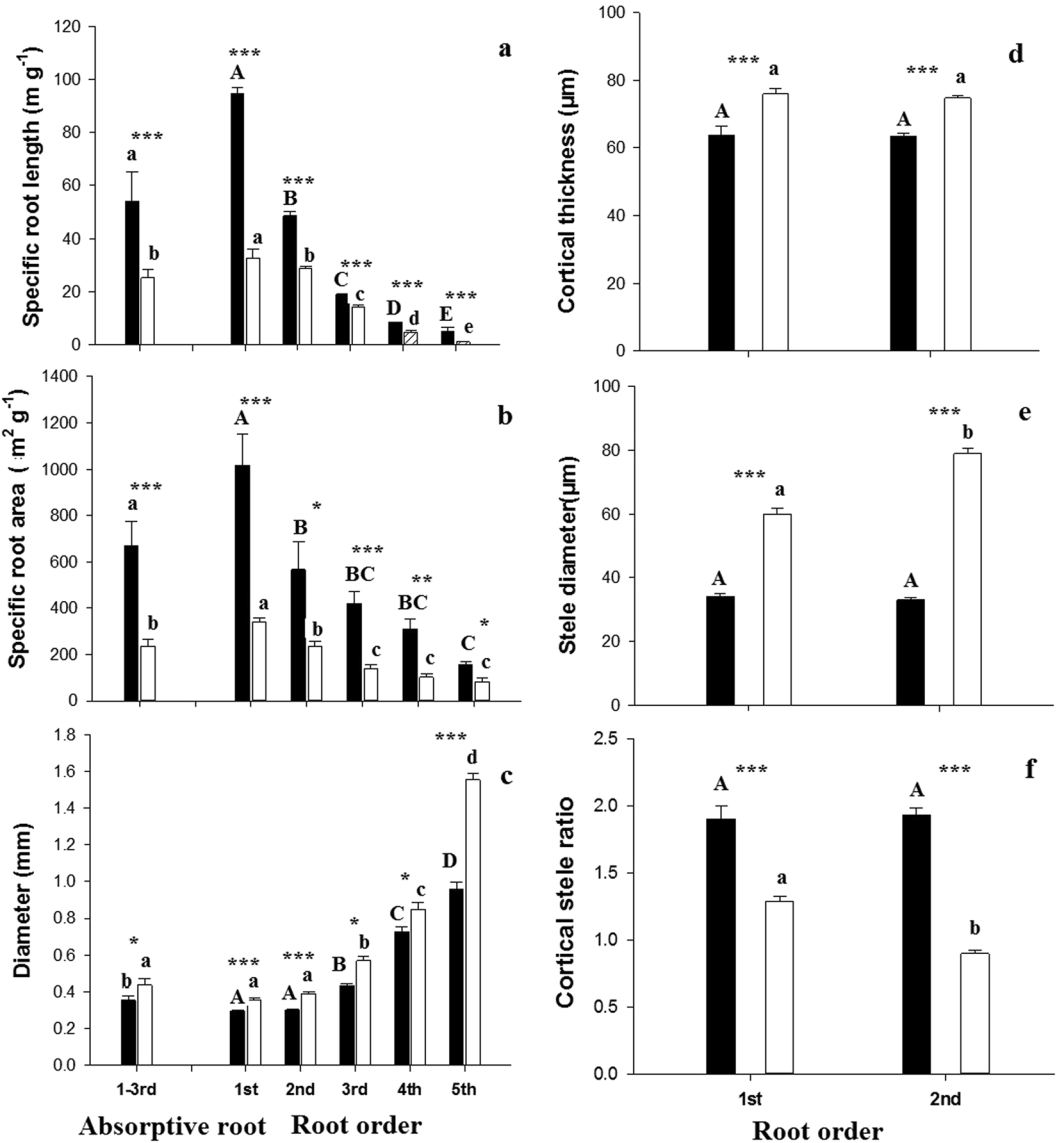
Root morphology (**a**–**c**) of individual root orders 1–5 and average of orders 1–3 (absorptive roots) and root anatomy (**d**–**f**) of order 1–2 of Manchurian walnut (black) and larch (white). Different upper case letters indicate significant differences between root orders of Manchurian walnut, lower case letters for larch (P < 0.05, Tukey HSD). Significant differences in a given root order class between species are marked with an asterisks (^*^P < 0.05; ^**^P < 0.01; ^***^P < 0.001, Tukey HSD; n = 10–16; mean ± SE).

Significant root architectural, morphological, and anatomical differences were found between species and within the branching hierarchies and when comparing the root orders of larch and Manchurian walnut (Table 1, Table S1, Figs 1–2). Length, biomass, diameter, cortical thickness, and stele diameter were significantly greater in larch than in Manchurian walnut root orders. In both species the length per root order decreased with increasing root order (Fig. 1a). While the biomass per root order decreased stepwise towards higher orders of larch, biomass was more equally distributed among root orders of walnut (Fig. 1b). In both species (monocultures), specific root length (SRL) and specific root area (SRA) decreased (Fig. 2a,b), although differences in SRA between orders were less pronounced in larch, while the diameter (Fig. 2c) increased with increasing root order. The stele diameter increased significantly from 1^st^ to 2^nd^ root order in larch only (Fig. 2e). The morphology of root orders 1–5 differed significantly across species: diameters of all root order (and thus cortex and stele diameters) were significantly greater in larch, while SRL and SRA were significantly greater in Manchurian walnut (Fig. 2a–c). The cortex:stele ratio was significant greater in 1^st^ and 2^nd^ order roots of Manchurian walnut than in larch grown in monoculture (Fig. 2f). Calculating the average morphology of root orders 1–3 (“absorptive roots”, i.e. taking the respective biomass frequencies into account) revealed that SRL and SRA were significantly greater in Manchurian walnut, although to a lesser extent than when comparing 1^st^ root orders directly, while root diameter were significantly greater in larch (Fig. 2a–c).

**Table 1:** Summary of the influence of monoculture species (Species), competition treatment: (mono or mix) and root order (1–5 Order) and their interactions on the root respiration, N, C/N ratio, root length, specific root area (SRA), specific root length (SRL), root diameter, root order biomass per branch, Cortical thickness, stele diameter and cortical stele ratio.

Source of variation	df	F	P	F	P	F	P	F	P	F	P	F	P
		Respiration		N		C/N		Length		SRL		SRA	
Treatment	1	**8.119**	**<0.047**	2.629	0.119	**1.656**	**<0.098**	**94.523**	**<0.001**	**47.192**	**<0.001**	2.362	0.132
Species	1	**478.743**	**<0.001**	**52.244**	**<0.001**	**82.058**	**<0.001**	**289.599**	**<0.001**	**41.798**	**<0.001**	**41.793**	**<0.001**
Order	4	**393.81**	**<0.000**	**248.977**	**<0.001**	**180.628**	**<0.001**	**681.321**	**<0.001**	**299.442**	**<0.001**	**44.458**	**<0.001**
Treatment* species	1	**3.826**	**<0.148**	0.003	0.96	**0.077**	**<0.62**	**6.417**	**<0.015**	2.659	<0.111	3.183	0.082
Treatment* order	4	1.053	0.392	0.278	0.891	1.113	0.097	**27.717**	**<0.001**	**5.859**	**<0.001**	0.757	0.559
Species* order	4	18.568	<0.021	1.945	0.122	**13.349**	**<0.001**	**76.939**	**<0.001**	**48.973**	**<0.001**	**7.315**	**<0.001**
Treatment* species* order	4	**2.829**	**<0.037**	**3.284**	**<0.020**	**5.239**	**<0.002**	**7.109**	**<0.001**	2.479	0.059	0.363	0.834
		**Diameter**		**Biomass/branch**		**Cortical thickness**		**Stele**		**Cortical stele ratio**			
Treatment	1	**1.445**	**<0.931**	**72.149**	**<0.001**	0.062	0.803	0.138	0.71	0.243	0.623		
Species	1	**36.595**	**<0.001**	**730.251**	**<0.001**	**55.555**	**<0.001**	**753.007**	**<0.001**	**131.951**	**<0.001**		
Order	4	**215.773**	**<0.001**	**81.998**	**<0.001**	**0.013**	**0.908**	**76.604**	**<0.001**	**6.115**	**<0.015**		
Treatment* species	1	1.563	0.218	**78.176**	**<0.001**	0.909	0.342	1.861	0.174	0.598	0.441		
Treatment* order	4	**2.383**	**<0.326**	**17.861**	**<0.001**	0.087	0.769	**2.236**	**0.137**	**0.037**	**0.848**		
Species* order	4	**4.674**	**<0.003**	**64.162**	**<0.001**	**2.528**	**0.114**	**60.046**	**<0.001**	**14.504**	**<0.001**		
Treatment* species* order	4	**1.871**	**<0.312**	**15.06**	**<0.001**	1.924	0.167	**0.103**	**0.748**	**1.048**	**0.308**		

Shown are the degrees of freedom (df), F and P values (P < 0.0001, P < 0.001, P < 0.05,) of the respective variables and variables with significant influence are printed in bold (P < 0.05).

**Figure 3:**
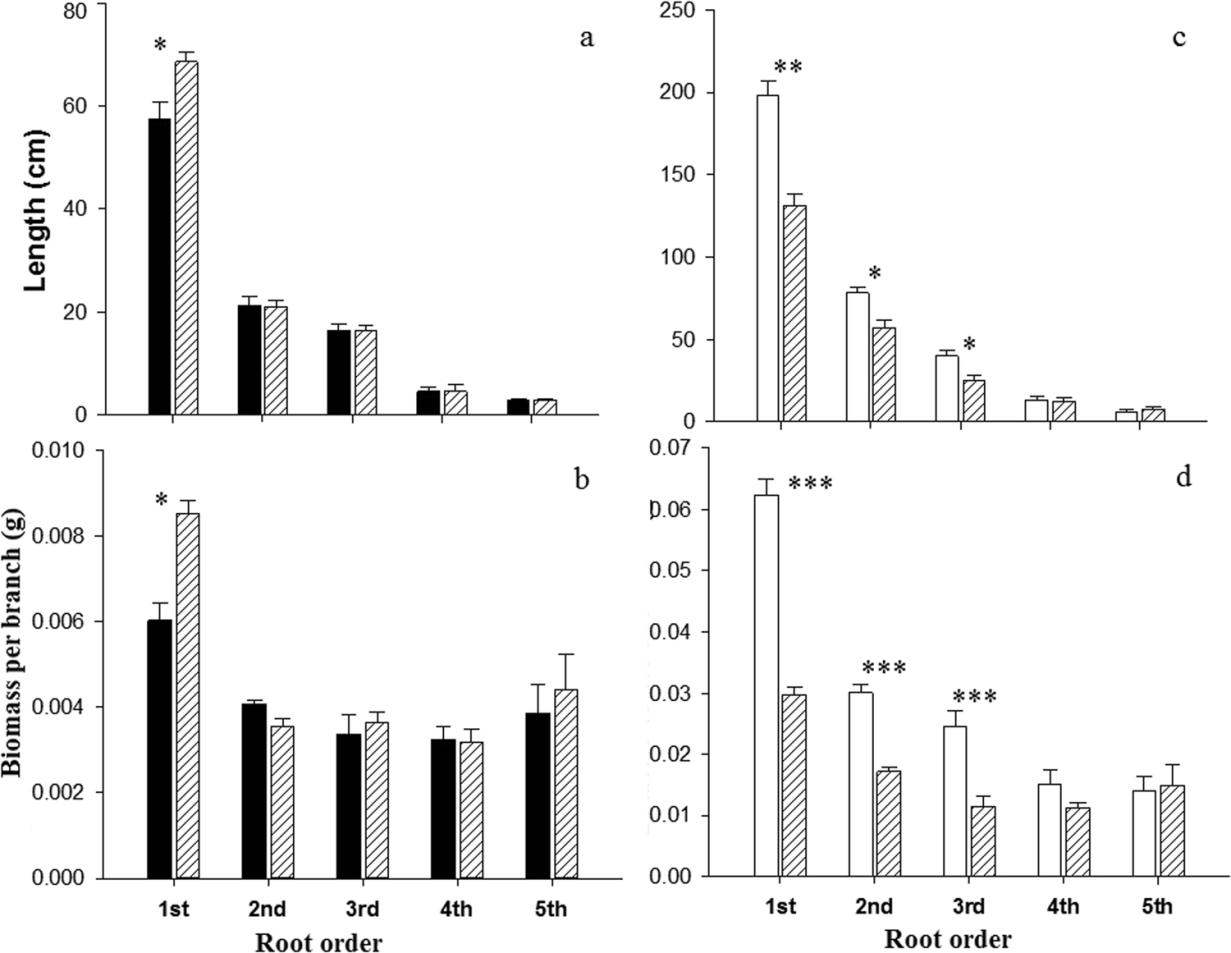
Root architecture of Manchurian walnut (**a**,**b**) and larch (**c**,**d**) root orders 1–5 in monocultures (walnut: black; larch: white) and mixed plantations (hatched). Please note differences in Y-axis scaling between species. Significant differences in a given root order classes per species between monoculture and mixed plantation are marked with an asterisks (^*^P < 0.05; ^**^P < 0.01; ^***^P < 0.001; Tukey HSD; n = 10–16; mean ± SE).

**Figure 4:**
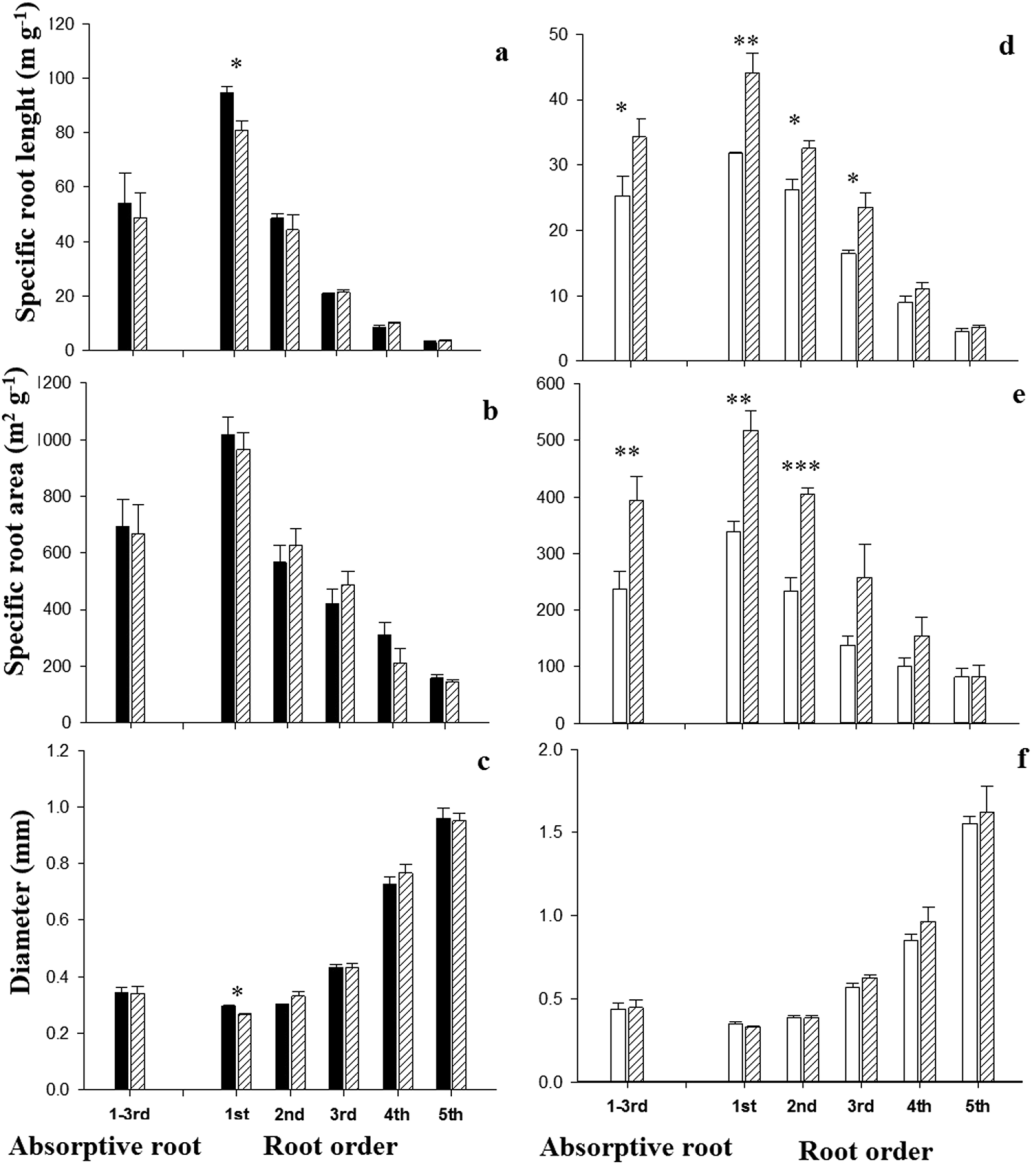
Morphology of Manchurian walnut (**a**–**c**) and larch (**d**–**f**) individual root orders 1–5 and average of orders 1–3 (absorptive roots) in monocultures (walnut: black; larch: white) and the mixed plantation (hatched). Please note differences in Y-axis scaling between species. Significant differences in a given root order classes per species between monoculture and mixed plantation are marked with an asterisks (^*^P < 0.05, Tukey HSD; n = 10–16; mean ± SE).

**Figure 5:**
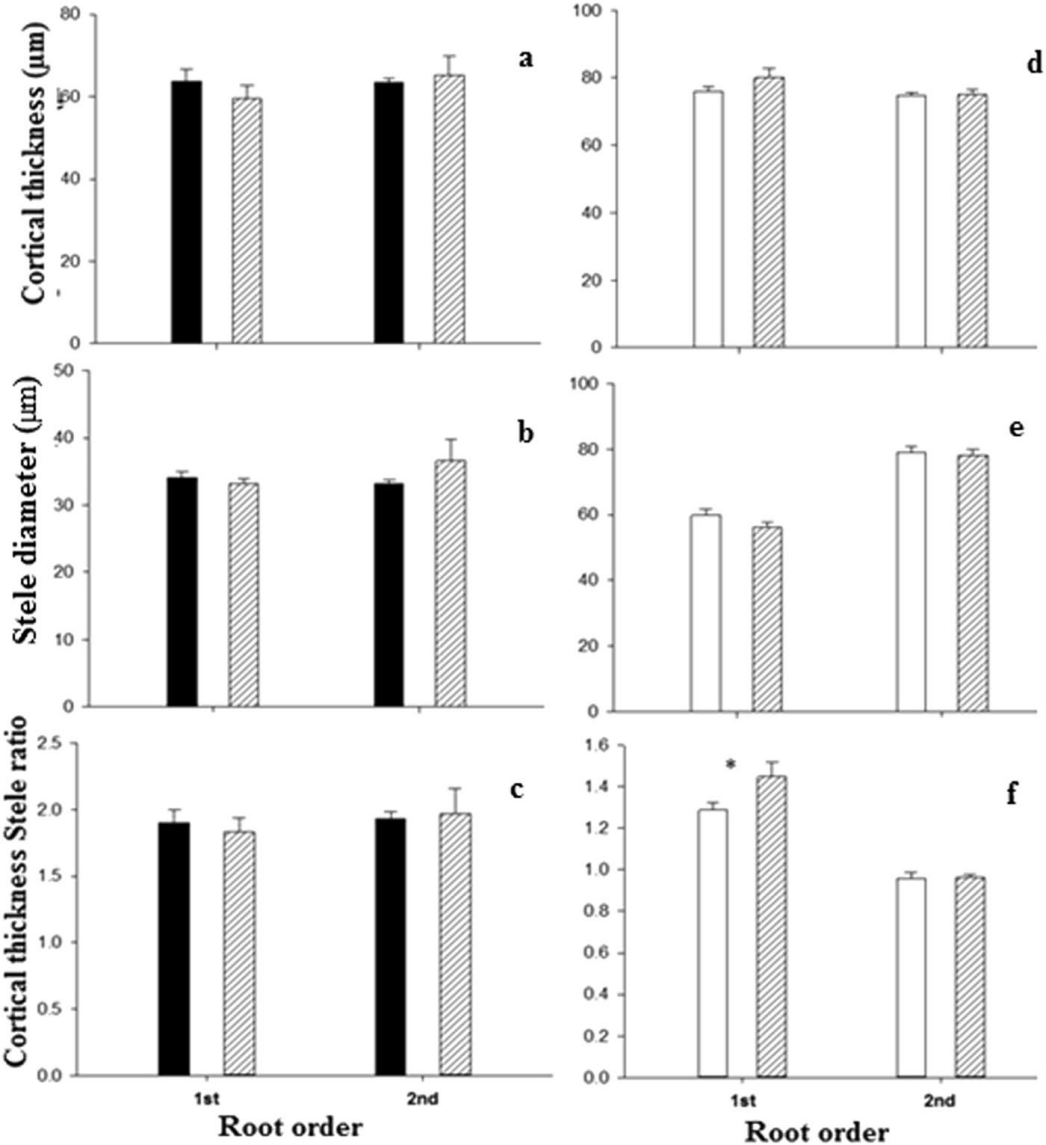
Root anatomy of Manchurian walnut (**a**–**c**) and larch (**d**–**f**) root orders 1–5 in monocultures (walnut: black; larch: white) and mixed plantations (hatched). Please note differences in Y-axis scaling between species. Significant differences in a given root order classes per species between monoculture and mixed plantation are marked with an asterisks (^*^P < 0.05; Tukey HSD; n = 30; mean ± SE).

The root architecture, morphology and anatomy of Manchurian walnut and larch changed between monocultures and mixed plantations (Table 1, Figs 3–5). The length and biomass of 1^st^ order roots (i.e. root tips) of Manchurian walnut were significantly greater in the mixed plantation than in the monoculture by +16% and +29%, respectively (Fig. 3a,b). In larch, length and biomass of root orders 1–3 were significantly greater in monoculture, for example by +30% and +49% for 1^st^ order roots, respectively (Fig. 3c,d). Specific root length (SRL, +14%) and root diameter (+8%) were significantly greater in the 1^st^ root order of Manchurian walnut in monoculture compared to the mixture (Fig. 4a,c). An increase in SRL/SRA accompanied by minor changes of root diameter indicates a decrease in tissue density. In larch, SRL and SRA were significantly greater (26% and 38% respectively) in the first three (SRL) or two (SRA) root orders under interspecific competition (Fig. 4d,e). While the anatomy of Manchurian walnut root orders 1 and 2 was unaffected by the competition type (Fig. 5a–c), the cortex:stele ratio of 1^st^ root order of larch significantly increased by 11% in the mixed plantation compared to the monoculture (Fig. 5f). In larch, the average SRL and SRA of root branches, including order 1–3, were significantly higher in mixed plantation as compared to the monoculture (Fig. 4d,e).

### Root respiration, C/N stoichiometry and mycorrhizal colonization

**Figure 6:**
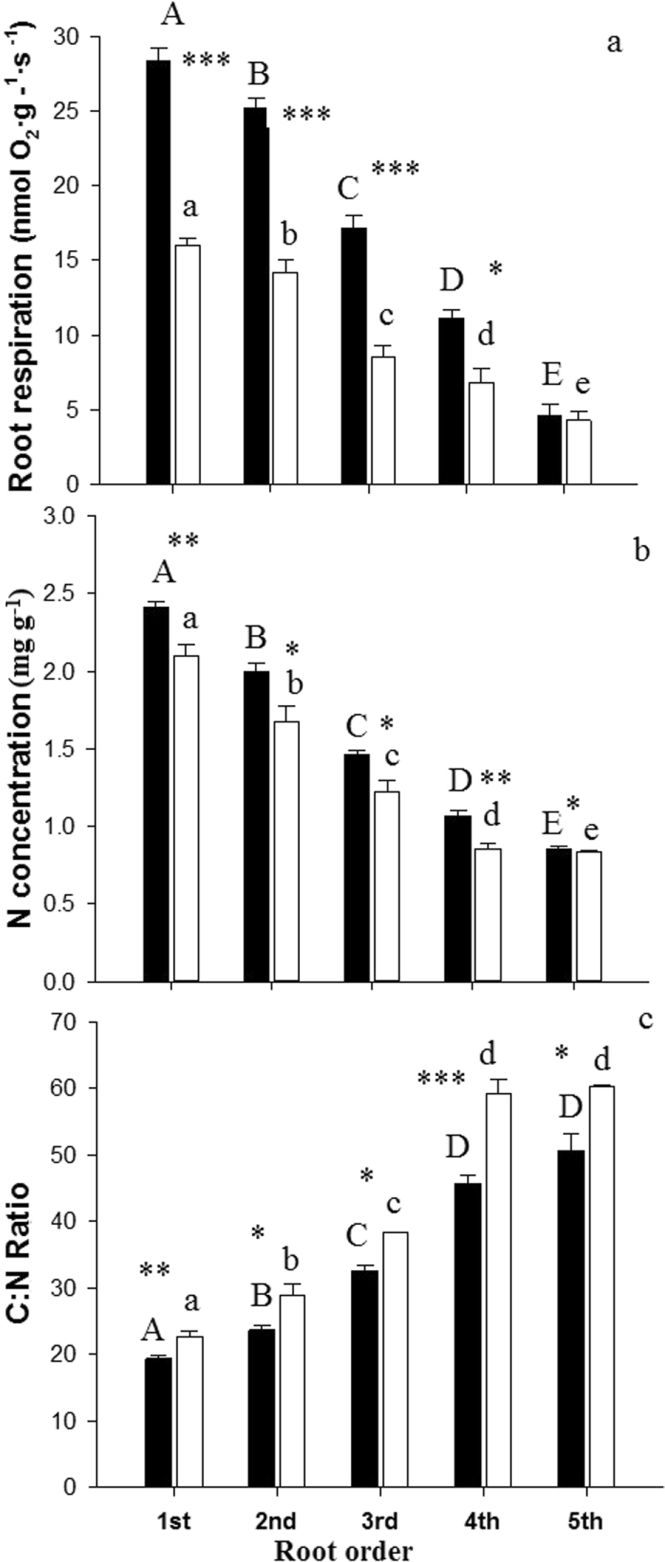
Root respiration (**a**) and C:N stoichiometry (**b**,**c**) of root orders 1–5 of Manchurian walnut (black) and larch (white). Different upper case letters indicate significant differences between root orders of Manchurian walnut, lower case letters for larch (P < 0.05, Tukey HSD). Significant differences in a given root order class between species are marked with an asterisks (^*^P < 0.05; ^**^P < 0.01; ^***^P < 0.001, Tukey HSD; n = 27; mean ± SE).

Significant root respiration and C/N stiochiometry differences were found between species (Table 1). The biomass-specific root respiration was significantly greater in root orders 1–4 of Manchurian walnut compared to larch and decreased with increasing order (Fig. 6a). Nitrogen concentrations across all five root orders were greater in Manchurian walnut than in larch (Fig. 6b); carbon:nitrogen ratios (C:N) significantly increased from root order 1 to 4 in both species (Fig. 6c).

**Figure 7:**
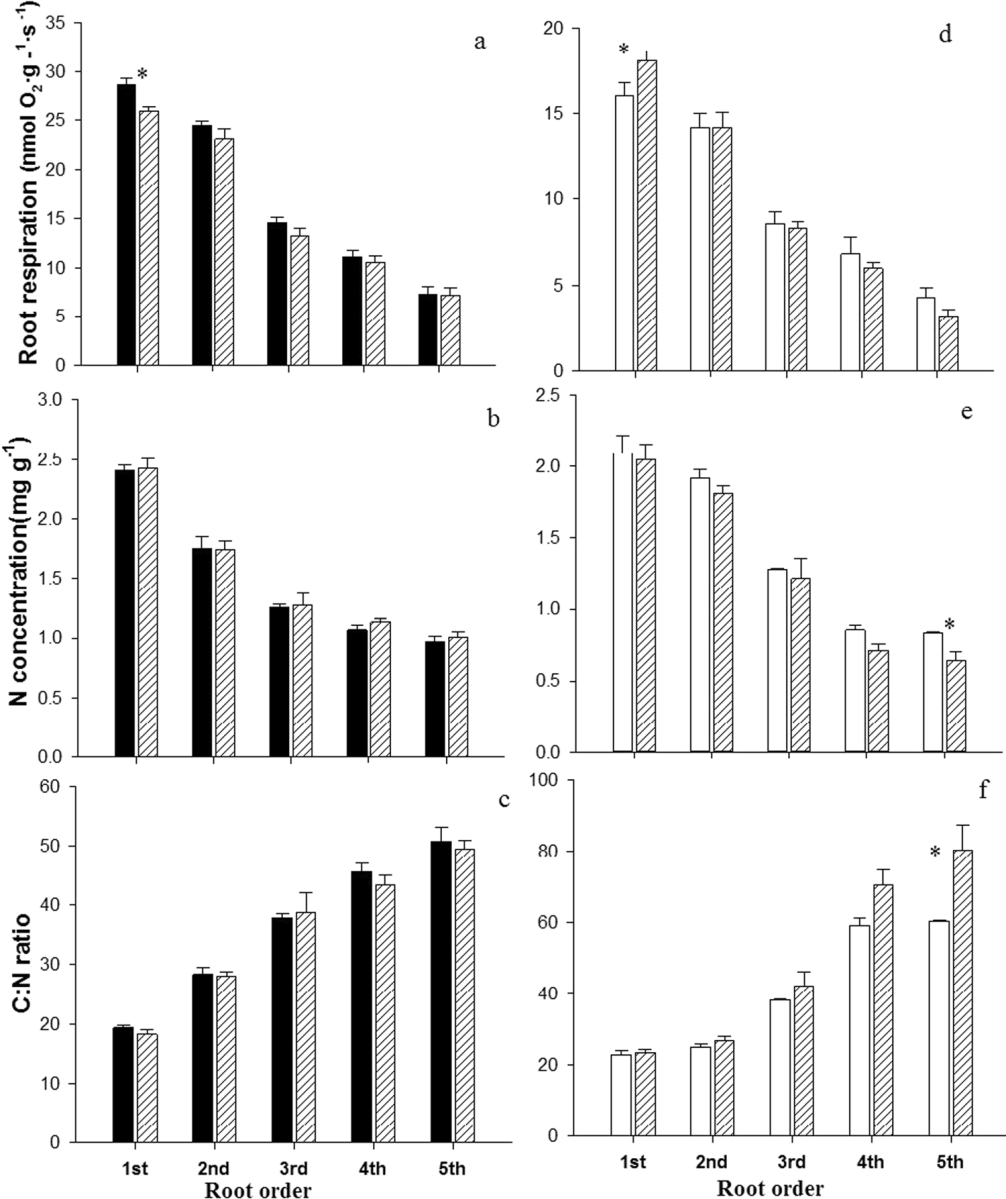
Root respiration (**a**,**d**) and C/N stoichiometry of Manchurian walnut (**a**–**c**) and larch (**d**–**f**) root orders 1–5 in monocultures (walnut: black; larch: white) and mixed plantation (hatched). Please note differences in Y-axis scaling between species. Significant differences in a given root order classes per species between monoculture and mixed plantation are marked with an asterisks (^*^P < 0.05, Tukey HSD; n = 10–15; mean ± SE).

Comparing monocultures and mixed plantation, the biomass-specific root respiration was significantly greater (9%) in the 1^st^ root order of Manchurian walnut in monoculture compared to mixture (Fig. 7a). In larch, the root respiration was significantly greater (12%) in the 1^st^ root order in mixture compared to the monoculture (Fig. 7d). Nitrogen concentrations and C:N ratios of Manchurian walnut root orders did not differ significantly between monoculture and mixed plantation (Fig. 7c). In larch, nitrogen concentration (−23%) and C:N ratio of the 5^th^ root order were significantly smaller/greater in the mixture, respectively (Fig. 7e,f).

**Figure 8:**
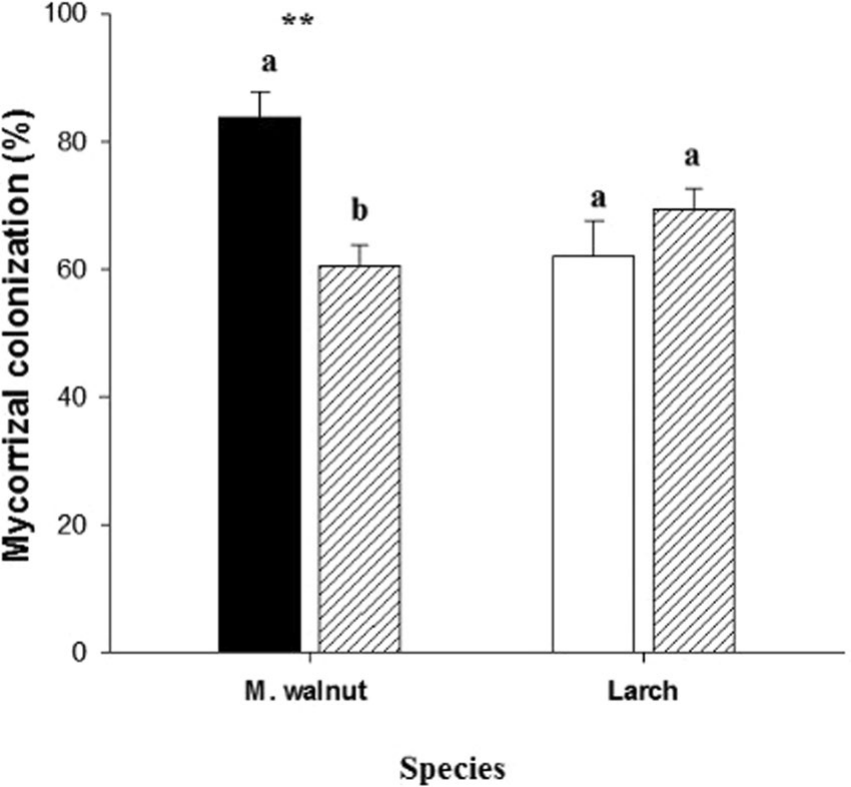
Mycorrhizal colonization rates of Manchurian walnut (AM) and larch (ECM) terminal root orders (first order) in monocultures (walnut: black; larch: white) and the mixed plantation (hatched). Significant differences per species between monoculture and mixed plantation are marked with an asterisks (^**^P < 0.01, Tukey HSD; n = 3–5; mean ± SE).

Arbuscular mycorrhizal colonization rates of terminal root orders of Manchurian walnut were significantly greater (+28%) in the monoculture compared to the mixed plantation; ecto-mycorrhizal colonization rates of larch root tips did not differ between monoculture and mixture (Fig. 8).

## Discussion

### Inter species differences in fine roots traits of Manchurian walnut and larch

Interspecific differences between tree root system largely affect tree growth by effects on the nutrient and water uptake capacity^36^ and the C balance^37^. For example, soil exploitation capacity and exploitation efficiency of the fine-root systems are largely determined by the root surface area/length per soil volume, root distribution within the soil horizons and specific root length (SRL)^38,39^. The uptake efficiency is determined by C costs related to the construction and maintenance of absorptive fine root surface^40^ and mycorrhizal symbionts^41^. In this study, the fine root systems of Manchurian walnut and larch grown in monocultures differed significantly in branching patterns (architecture) and root order-specific morphological, anatomical, chemical and physiological traits. The data on fine root length and biomass of root orders indicates that larch trees, growing under the given edaphoclimatic conditions, have generally more extensive (in terms of length and biomass) fine root branches (order 1–5) than Manchurian walnut trees. In addition, larch root branches feature a much larger proportion of lower, absorptive root orders (1–3) than Manchurian walnut where length and biomass distribution evidence a more homogeneous contribution of individual orders to the architecture of root branches. Both the higher biomass and length of lower (i.e. first two or three orders), absorptive root orders should thus benefit larch’s absolute capacity to forage resources^31,42^, although the uptake capacity per root surface (of different orders) remains an unknown factor for nutrients with active, transporter-based uptake mechanisms and potentially also for water/nutrients taken up by mass flow^43^. Variations in root diameter, but also cortex thickness and stele diameter were previously reported to be strongly phylogenetically determined^44^. Individual larch root orders hold slightly larger diameter but distinctly reduced SRL and SRA values compared to Manchurian walnut root orders, indicating (together with the larger stele) also a higher tissue density of individual larch root orders. Larger diameter and lesser SRL/SRL are often related to a lower efficiency of a species in building root length and surface area for exploration and uptake of resources^45^.While uncertainties remain in term of root longevity^46^, these finding indicate that Manchurian walnut trees possesses a more efficient root system than larch in terms of structural C utilized for building root surface/length. Previous studies showed similarly that conifers typically have thicker diameter roots and lower SRL than coexisting deciduous angiosperms^38,47^. The large root order specific morphological differences in the studied species are partially “balanced” by the divergent frequency of root orders in both species (as evidenced by biomass and length, Fig. 1). However, while differences are reduced when averaging the traits of absorptive root orders (e.g. 2-fold differences for SRL of root branches 1–3 compared to a 3-fold difference in SRL of 1^st^ order roots), SRA and SRL of Manchurian walnut root branches (i.e. as average of orders 1–3) are still significantly greater than of larch absorptive root branches. In contrast to the morphological traits, the lower maintenance respiration of larch (in root orders 1 to 3), potentially related to larger diameter and lower N contents^32,48^, might serve as an indication of a greater C use efficiency of individual larch roots compared to Manchurian walnut. Root respiration was previously shown to constitute a major component of plant C budgets^49,50^, especially under suboptimal growing conditions^51,52^.

Acquisitive (fast growing) species often possess relatively small-diameter, high specific root length (SRL), fast-growing fine roots with short lifespan, high N content, and high rates of respiration and nutrient acquisition in comparison to conservative (slow growing) conifer species^19,53^. While we found the hypothesized, intrinsic differences of root traits between the two species^54^, we could not link the root traits in monocultures to the (aboveground) growth performance of both species. Under the given environmental conditions and in monocultures, larch trees possessed a higher aboveground growth than Manchurian walnut during the last three decades. According to previous findings, it is reasonable to speculate that auto-inhibitory effects by juglone in Manchurian walnut monocultures^55,56^ might have dominantly contributed to the growth depression. We suggest also that the rather a typical root branching pattern of Manchurian walnut, compared to other trees species featuring less even proportions of root orders^32,57^ might be a direct consequence of the juglone concentration in the soil. Further studies are thus urgently needed to relate root traits of Manchurian walnut to different levels of Juglone present in the soil; these studies should also include information on root system sizes and architecture to calculate realistic, species-specific traits of root branches^58^ and to identify important species-specific traits of the whole (fine) root systems.

### Influence of interspecific competition on fine root traits

Interspecific competition in the mixed plantation resulted in reduced growth of larch trees while Manchurian walnut trees tended to show greater DBH and increased height growth in mixtures compared to monocultures. While this is likely an effect of both above- and belowground interactions between species^8,59,60^, it is well documented that walnut species release juglone into the soil which inhibit the growth of neighboring plants including seedlings of the own species after a certain accumulation^61,62^. Thus, in the following we will focus on root traits affected by interspecific competition. Root systems are a quickly responding plant organ that is influenced by a variety of dynamic factor including internal species heredity character^54^ and external conditions e.g., nutrient availability, soil properties and non-toxic signals/toxic allochemicals released by neighboring plants^63^. Numerous previous studies have reported on the influence of competing roots on the (root) biomass and biomass distribution of neighboring species^64^. While root competition intensity is easiest determined by plant responses (e.g. effects on yield, relative growth rate, survival etc.) and potentially best measured by comparing soil resource availability in the presence and absence of competing roots^65^, few previous studies have reported detailed effects of different competitive environments on root (order) traits of woody species^54,66^.

Especially root architectural and morphological traits are believed to be important indicators of resources acquisition capability^29,67^ and construction cost^68,69^. In this study, the length and biomass and of larch root orders 1–3 were significantly greater, while the SRL and SRA of the first three/two root orders were significantly lesser in monoculture as compared to larch in the mixed stand. In contrast, plasticity of these traits under interspecific competition was limited in Manchurian walnut to the first root order (i.e. root tips). It is noteworthy that especially the terminal, absorptive root orders of the fine root system possessed a different morphology under intra- vs. interspecific competitive environments, while the higher order (“transport” fine roots) did not vary in most assessed traits. However, given that root competition affects the availability of soil resources, this is coherent with earlier finding reporting a greater morphological plasticity of absorptive root orders to changes in soil moisture^70,71^, nutrient availability^72,73^, or heavy metal/salt stress^43^. The reduced frequency of terminal root orders in larch may be at least partially related to a direct growth inhibition by juglone, as shown previously for corn root systems^74^. In both tree species, modified tissue densities likely caused the changes in SRL/SRL because root diameters were less plastic. In larch, the change in tissue density of 1^st^ root orders (root tips) seem to be largely based on the greater cortex:stele ratio—the cortex holding a lower tissue density than the stele. Kong *et al*.^75^ proposed earlier that the allometric relationship of cortex and stele sizes reflects the relative importance of resources absorption and transportation in woody species; as such, the increased cortex of larch may be an indication of increased nutrient uptake capacity (e.g. by increasing exudation capacity^76^. However, we also deem it possible that an increased cortex holds advantages when facing high juglone concentrations, similar to previous findings of increased cortical tissue thickness under excess NaCl salinity^77^; however, this and the growth/branching inhibiting effect of juglone on larch root systems need further examination.

In this study, interspecific root competition significantly increased the respiration of root tips in larch and decreased it in Manchurian walnut compared to the respective monocultures. Meier *et al*.^78^ have earlier shown a substantial increase in root respiratory O_2_ uptake when pea plants were exposed to non-self competitors. An increase in respiration may indicate a more active root system^40,79^ and Volder *et al*.^30^ suggested further that high root respiration rates may be an indicator of a higher competitive ability if accompanied by increased nutrient uptake capacities. However, increased root respiration rates can also be a direct driver of competition affecting soil CO_2_ concentrations and thus inhibiting growth of neighboring roots^80^. The increased respiration in larch root tips in mixture could also be related to their increased cortical tissue^81^, or to higher respiratory costs for metabolic defense and repair mechanisms^82–84^. Concerning the latter, the decrease in root respiration in Manchurian walnut could then be related to the reduced concentration of toxic juglone in mixed plantation soil^85–87^. However, contrasting results exist on the effect of juglone on O_2_ consumption rates of other species, reporting to either affect or not affect respiration rates of soybean and corn^74,88^, thus, further work is necessary to clarify the situation for the given species.

The changes in mycorrhizal infection rates under interspecific competition are likely related to different nutrient availabilities, especially P, in the mixed/monoculture production systems. The microbial biomass (via PLFA) decreases dramatically as the juglone concentration increased in Manchurian walnut stand^15^. Thus, a potentially higher microbial biomass in monocultures of larch might have facilitated the plant availability of P and other nutrients. Both arbuscular and ectomycorrhizal symbionts have previously been shown to be especially enhance P uptake^89,90^; however, especially ectomycorrhizal tree species invest more in mycorrhizal maintenance and hyphae proliferations to maximize nutrient foraging^91,92^. In Manchurian walnut, the higher P availability in mixed plantation compared to monocultures might thus result in a decreased investment into mycorrhizal symbionts, while the ectomycorrhizal colonization rate in larch tended to be increased due to the lower availability of P (but potentially also other nutrients) in mixture compared to larch monocultures.

In general, larch’s absorptive root orders possessed a greater plasticity under interspecific competition than the individual root segments of Manchurian walnut. We speculate, that the greater plasticity of larch, especially in terms of branching patterns, root morphology and anatomy, could have been either caused by the relative conservative morphology of this species in terms of C use efficiency (low SRL, SRA, greater root diameter)^40^, or is a direct consequence of the accumulated juglone in the shared soil^74^. Previously, greater SRL and SRA values were reported in mixed species forests relative to monocultures, likely increasing the competitive ability^28,93^. Conversely, the limited plasticity of Manchurian walnut root orders under interspecific competition, compared to larch, might be due to the increased nutrient availability in mixture with larch, as indirectly evidenced by decreased AMF colonization rates, SRL, and respiration rates of 1^st^ order roots. However, it is also reasonable to speculate that Manchurian walnuts competitive “strategy” may be rather based on interference competition *via* juglone exudation and not exploitative competition—resulting in a lower plasticity in uptake-related root traits. Further studies are necessary to separate the direct allopatric effects of juglone from resource competition effects on fine root traits, potentially using an inoculation with *Pseudomonas* sp. strains to degrade the exudated juglone rapidly^94^ and/or establishing a fertilization gradient.

## Material and Methods

### Site description

This experiment was conducted at the Maoershan experimental station (45°16′ N, 128°34′ E), located in the temperate forest region of the Heilongjiang Province, NE China. The climate is a continental monsoon climate featuring a strong monsoon in the spring, a warm and humid summer, and a dry and cold winter. The average annual air temperature is 2.8 °C; average temperatures in January and July are -19.6 °C and 20.9 °C, respectively. The frost-free period is 120 to 140 days-long. The annual averages of rel. humidity, precipitation, and potential evapotranspiration are 70%, 724 mm a^−1^, and 1094 mm a^−1^, respectively. The parent material is granite bedrock and soils are Boric Luvisols^95^; the bulk soil density (0–10 cm depth) is 0.83 g cm^−3^ ^96^; further soil characteristics are listed in Table 2.

**Table 2:** Summary of stand and soil characteristics of three plantations used in this study: monocultures (Mono) of *Larix gmelinii* and *Juglan mandshurica* and their mixed plantation (Mix) in NE China.

Plantation type	Species	Stand characteristics			Soil characteristics					
		Density (n ha^−1^)	DBH (cm)	Height (m)	pH	OM (g kg^−1^)	Total N (g kg^−1^)	Hydrolytic N (mg kg^−1^)	Total P (g kg^−1^)	Available P (mg kg^−1^)
Mono	Larch	1284	80.7	13.2	4.7	6.29	5.48	409.2	0.56	11.5
Mono	Walnut	1500	54.9	11.8	4.9	6.63	5.72	451.3	0.59	7.5
Mix	Average	1380	—	—	5.1	6.41	6.47	524.1	0.61	8.5
Mix	Larch	735	41.7	13.7	—	—	—	—	—	—
Mix	Walnut	645	65.4	13.0	—	—	—	—	—	—

Stand characteristics given are tree density, diameter at breast height (DBH) and tree height, soil characteristics (0–10 cm soil depth without litter) given are pH (H_2_O), organic matter (OM), total and hydrolytic Nitrogen (N), and total and available Phosphorous (P) (mean; n_DBH/Height_ = 13–15, n_Soil_ = 3–5).

Larch (*Larix gmelinii* Rupr.) and Manchurian walnut (*Juglan mandschurica*) are dominant species in natural forests, and key species used for plantations in Northeast China. The two species contrast in taxonomy (gymnosperm *vs*. angiosperm), and type of mycorrhizal symbionts (larch: ectomycorrhizal; Manchurian walnut: arbuscular)^97^. Due to their economic importance, large monocultures and mixed plantations of the two species were established in the region in 1987; 2-year-old seedlings were planted in a 1.5 m × 1.5 m grid (between rows × within rows) in all three stands. Line mixing (i.e. three rows of Manchurian walnut followed by five rows of larch) was used in the mixed-species plantation. Stand characteristics in the year 2017 are listed in Table 1; differences in tree densities are caused by mortality. Three adjacent stands, two monocultures of larch and Manchurian walnut (intraspecific competition), respectively, and a mixed plantation (interspecific competition) consisting of the two species, were selected for this study. Plots were located on a SW-facing slope (approx. 13°) at 450–500 m a.s.l. In monocultures, three subplots were established at random locations (distance > 10 m) in-between rows; in mixed-plantation, subplots were located between rows holding larch and Manchurian walnut trees respectively. At each subplot, samples were taken at three locations; in monocultures the locations were randomly selected. In mixed stands, the locations were chosen by following roots from opposing larch and Manchurian walnut boles, samples were taken at locations were root systems of both species overlapped in the top soil. In total nine locations were sampled per stand type.

### Root architecture and morphology

Nine soil monoliths (15 cm × 15 cm) per stand were carefully sampled at 0–10 cm depth at the end of July after removing the litter from the soil surface, following the method by Guo *et al*.^97^. Root samples were stored in plastic bags after coarsely shaking off the soil and transported to the laboratory (8 °C). In the lab, root samples were rinsed in water and adhering soil particles were carefully removed with small brushes; dead roots were discarded. Subsequently, 10–15 intact root branches per soil monolith and species were dissected into five branching orders^32,33^, first order roots being defined as the root tips. Lateral roots with ≤4 root orders and directly originating from branching orders ≥6 as well as root segments of orders ≥6 were discarded. Root order samples were individually imaged with a flatbed scanner (Expression 10000XL with transparency unit, Epson, Japan; 600 dpi, gray-scale) and analyzed with the software WinRhizo 2004b (Regent Instruments, Québec, Canada) for morphological traits, including total root length (cm), surface area (cm^2^) and average root diameter (AD; mm). The root segments were dried to constant mass (65 °C) and dry mass was determined to an accuracy of ±0.1 mg (Sartorius BT 125D, Göttingen, Germany). Specific root area (SRA; m^2^ g^−1^) and specific root length (SRL; m g^−1^) were calculated by dividing the surface area/length by the dry mass. Root architecture traits calculated were length (cm) and biomass (g) per root order^98,99^. Average SRL, SRA and AD for root orders 1–3 were calculated by multiplying the respective values of root orders 1 to 3 (i.e. “absorptive” root segments) with their respective biomass frequency (1–3) in individual root branches.

### Root respiration and root chemistry

Small batches of roots were sampled as describes above and immediately transported to the lab where roots were rinsed carefully with de-ionized water to remove adhering soil particles. Three root branches per soil pit were sectioned into root orders as described above; dead roots were discarded. Root respiration measurements were initiated within 40–50 min after root sampling and were completed within 4 hours after sample collection. Individual root order samples (approx. 0.5 g fresh weight) were placed in vented chambers and equilibrated for 20 min to 18 °C; root desiccation was avoided by placing a piece of moist paper towel into each chamber until measurements started. Root respiration was approximated by measuring the O_2_ consumption for 20 min, using gas-phase O_2_-electrodes (Hansatech, Norfolk, UK), connected to a circulating water bath (18 °C)^100^. After measurements, the samples were dried (65 °C, 72 h) and weighed (±0.01 mg). Root respiration was calculated as nmol O_2_ g^−1^ s^−1^ by dividing the respiration rate by the corresponding dry weight. Per soil pit, three respiration measurements per root order and species were conducted, resulting in 27 respiration measurements per root order and species in both mono and mixed plantations (n = 27; 540 measurements in total). The dried samples were individually ground and homogenized; anElemental Analyzer (Vario MACRO; Elementar, Langenselbold, Germany) was utilized to determine N and C concentrations (%). C/N ratios were calculated.

### Root anatomy and Mycorrhizal colonization

Five randomly selected root branches per species and competition situation (sampling scheme see above) were gently washed in de-ionized water and immediately fixed in Formalin-Aceto-Alcohol (FAA) solution (90 ml 50% ethanol, 5 ml 100% glacial acetic acid, 5 ml 37% methanol) and stored in a refrigerator (4 °C). In the lab, thirty segments each of 1^st^ and 2^nd^ order roots were stained with safranin-fast green, dehydrated in 70, 85, 95 and 100% EtOH, and embedded in paraffin. Cross sections (8-μm) were prepared with a microtome and photographed under a compound microscope (BX-51; Olympus Corporation, Tokyo, Japan). Cortex thickness (µm) and stele diameter (µm) were measured^97^; the cortex:stele ratio was calculated.

The colonization of walnut and larch roots with arbuscular (AM) and ecto (ECM) mycorrhizal fungi, respectively, wasinvestigated on 1^st^ order roots (root tips). For determining the AM colonization rate, walnut root segments were bleached with 10% KOH (90 °C, 50 min), rinsed in distilled water, and acidified (3.7% HCl, 15 min, room temp.). After repeated cleaning with distilled water, the roots were stained for 90–120 s in lactophenol-blue (1 g L^−1^, pH 2.3; Merck)^101^. To remove unspecific colorant from the plant tissue, the roots were incubate for 60 min in an acidic glycerin solution (50 ml glycerin, 45 ml H_2_O, 5 ml 1% HCl) at room temperature; the solution was renewed after 30 min. The roots were then stored in 50% glycerine until mounting on microscope slides as squash preparation and AM colonization rate determination^102^. The infection rate of ECM on larch roots was determined by counting the root tips with a hyphae mantel under a stereomicroscope (40×); >100 tips were screened per larch root branch.

### Statistical analysis

The statistical analyses were carried out with the software IBM SPSS 21.0 and Microsoft Excel 2010. Sigma Plot 12.5 (Systat software Inc.) was used to draw the figures. The effect of species, treatment and root order were analyzed by analysis of variance (ANOVA). Further, subsequent pair-wise comparisons were performed to identify the differences in the root traits using Tukey’s HSD test. Normality of data distribution was checked with a Shapiro-Wilk test. All data is displayed as mean ± standard error (SE). All statistical relationships were considered significant at *P* < 0.05.

## Electronic supplementary material

Table S1
