# Supplementary material for: Root order-based traits of Manchurian walnut & larch and their plasticity under interspecific competition
Source: Sci Rep. 2018 Jun 29;8:9815. doi: 10.1038/s41598-018-27832-0 (PMC6026125; doi:10.1038/s41598-018-27832-0)
Supplement: Supplementary file 1 — Table S1 [file 41598_2018_27832_MOESM1_ESM.docx]

Root order-based traits of Manchurian walnut & larch and their plasticity under interspecific competition

Salahuddin, Boris Rewald, Muhammad Razaq, Yang Lixue*, Ji Li, Farmanullah Khan, Zhang jie

**Table.S1** ANOVA of the influence of monoculture species (Species), competition treatment: (mono or mix) and root order (1–5 order) and their interactions on the root respiration, N, C/N ratio, root length, specific root area (SRA), specific root length (SRL), root diameter, root order biomass per branch, Cortical thickness, stele diameter and cortical stele ratio.

| Source of variation | dF | F | P | | F | P | F | P | F | P | F | P |  |  |
| --- | --- | --- | --- | --- | --- | --- | --- | --- | --- | --- | --- | --- | --- | --- |
|  |  |  |  | |  |  |  |  |  |  |  |  |  |  |
|  |  | Respiration | | | N | | C/N | | Length | | SRL | | SRA | |
| **Treatment** | 1 | **8.119** | **<0.007** | | 2.629 | 0.119 | **12.656** | **<0.001** | **94.523** | **<0.001** | **47.192** | **<0.001** | 2.362 | 0.132 |
| **Species** | 1 | **478.743** | **<0.001** | | **52.244** | **<0.001** | **82.058** | **<0.001** | **289.599** | **<0.001** | **41.798** | **<0.001** | **41.793** | **<0.001** |
| **Order** | 4 | **393.81** | **<0.001** | | **248.977** | **<0.001** | **180.628** | **<0.001** | **681.321** | **<0.001** | **299.442** | **<0.001** | **44.458** | **<0.001** |
| **Treatment * species** | 1 | **7.826** | **<0.008** | | 0.003 | 0.96 | **5.877** | **<0.020** | **6.417** | **<0.015** | 2.659 | <0.111 | 3.183 | 0.082 |
| **Treatment * order** | 4 | 1.053 | 0.392 | | 0.278 | 0.891 | 2.113 | 0.097 | **27.717** | **<0.001** | **5.859** | **<0.001** | 0.757 | 0.559 |
| **Species * order** | 4 | **18.568** | **<0.00** | | 1.945 | 0.122 | **13.349** | **<0.001** | **76.939** | **<0.001** | **48.973** | **<0.001** | **7.315** | **<0.001** |
| **Treatment * species * order** | 4 | **2.829** | **<0.037** | | **3.284** | **<0.020** | **5.239** | **<0.002** | **7.109** | **<0.001** | 2.479 | 0.059 | 0.363 | 0.834 |
|  |  |  |  | |  |  |  |  |  |  |  |  |  |  |
|  |  | Diameter | | | Biomass / branch | | Cortical thickness | | Stele | | Cortical stele ratio | |  |  |
| **Treatment** | 1 | **14.45** | | **<0.001** | **72.149** | **<0.001** | 0.062 | 0.803 | 0.138 | 0.71 | 0.243 | 0.623 |  |  |
| **Species** | 1 | **36.595** | | **<0.001** | **730.251** | **<0.001** | **55.555** | **<0.000** | **753.007** | **<0.001** | **131.951** | **<0.001** |  |  |
| **Order** | 4 | **215.773** | | **<0.001** | **81.998** | **<0.001** | **0.013** | **0.908** | **76.604** | **<0.001** | **6.115** | **<0.015** |  |  |
| **Treatment * species** | 1 | 1.563 | | 0.218 | **78.176** | **<0.001** | 0.909 | 0.342 | 1.861 | 0.174 | 0.598 | 0.441 |  |  |
| **Treatment * order** | 4 | **6.383** | | **<0.001** | **17.861** | **<0.001** | 0.087 | 0.769 | **2.236** | **0.137** | **0.037** | **0.848** |  |  |
| **Species * order** | 4 | **4.674** | | **<0.003** | **64.162** | **<0.001** | **2.528** | **0.114** | **60.046** | **<0.001** | **14.504** | **<0.001** |  |  |
| **Treatment * species * order** | 4 | **5.07** | | **<0.002** | **15.06** | **<0.001** | 1.924 | 0.167 | **0.103** | **0.748** | **1.048** | **0.308** |  |  |

Shown are the degrees of freedom (df), F and P values (P<0.0001, P <0.001, P <0.05,) of the respective variables and variables with significant influence are printed in bold P<0.05).
